# Polyphenolic and Physicochemical Properties of Simple-Spined Num-Num (*Carissa edulis*) Fruit Harvested at Ripe Stage of Maturation

**DOI:** 10.3390/molecules24142630

**Published:** 2019-07-19

**Authors:** Fulufhelo P. Makumbele, Malcolm Taylor, Marietjie Stander, Tonna A. Anyasi, Afam I.O. Jideani

**Affiliations:** 1 Department of Food Science and Technology, School of Agriculture, University of Venda, Private Bag X5050, Thohoyandou 0950, South Africa; 2 Mass Spectrometry Unit, Central Analytical Facilities, Stellenbosch University, Private Bag X1, Matieland 7600, South Africa

**Keywords:** *Carissa edulis*, phytochemicals, antioxidant activity, physicochemical properties, free radicals, ripening stage

## Abstract

Wildly grown in most regions of the world, *Carissa edulis* is a highly underutilised fruit with significant antioxidant characteristics. The phyto and physicochemical properties of *C. edulis* berries at different stages of ripening are evaluated in this work. Total flavonoids (TF), total phenolic content (TPC) and antioxidant activity were determined spectrophotometrically, while concentration of polyphenols was determined using liquid chromatography coupled to diode array detection and electrospray ionization mass spectrometry. Results showed that antioxidant activity was lowest (18.36 ± 0.12 mmol TE/g) in RS3 and decreased with TPC upon increased ripening. Conversely, TF increased with ripening progression with TF found to be highest in RS3 (5.92 ± 0.03 mg CE/g). Identified phenolic acids in *C. edulis* were quinic acid, protocatechuoyl-hexose, neochlorogenic acid, chlorogenic acid, cryptochlorogenic acid and dicaffeoylquinic acid. Identified flavonoids included rutin, catechin, procyanidin dimer, procyanidin trimer, quercetin-3-*O*-glucosyl-xyloside, quercetin-3-*O*-robinobioside, quercetin-3-*O*-glucoside and quercetin-3-OH-3-methylglutaryl-glucoside. Physicochemical properties of *C. edulis* varied among samples with sugar/acid ratio of *C. edulis* ranging from 25.70 for RS1 to 50.36 for RS3. Ripening stage of *C. edulis* undoubtedly affects the phyto and physicochemical properties of *C. edulis*.

## 1. Introduction

**Figure 1:**
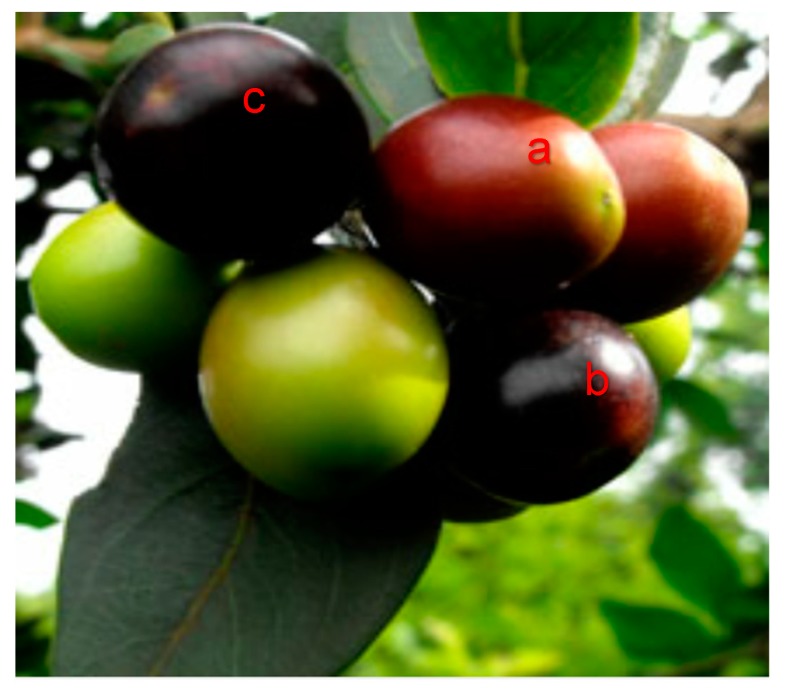
*Carissa edulis* fruits at different ripening stages used for the study. (**a**) First stage of ripening; (**b**) second stage of ripening and (**c**) last stage of ripening. Source: Makumbele [4].

*Carissa edulis* is a much branched spiny evergreen shrub or small tree, usually multi-stemmed, often scrambling up to 6 m tall and forming a dense canopy. All parts of the plant release white, non-toxic milky latex. Young branches are green, smoothly covered with minute hair, but older branches and stems become light brown and corky with deep cracks. The plant is armed with rigid spines up to 70 mm long and nearly always simple, not forked as with other species [1]. Leaves are simple, opposite, leathery, dark green above and paler below, while fruits are green, dark red and purple as ripening progresses. Fruits are also fleshy, ovoid, 6–11 mm in diameter, red to purplish black berries and two- to four-seeded (Figure 1). In the works of Muthsinyalo and Malatji [2], *C. edulis* is reported to occur in bush-veld, often in riverine vegetation, on termite mounds and is common in deciduous to evergreen woodland. Distribution of the fruit in Africa is from Senegal to East Africa and from Mpumalanga to Limpopo in South Africa [2]. Globally, the fruit is cultivated in Thailand, India and Indian Ocean Islands. *Carissa edulis* is generally referred to as a berry due to its small nature, seedless/small seed and its ability to be eaten whole [3].

Harvest maturity and ripening stage of maturity are some factors that may lead to changes in sensory and nutritional qualities of *C. edulis*. Moreover, there has been a constant increase in popularity and interest regarding research of all kind of fruits, including berries, in the last few decades, as they are reported to be a good source of bioactive compounds [5]. Recent interest in food phenolics has greatly increased because of the antioxidant and free radical-scavenging abilities associated with phenolics and their potential effects in human health [6]. Bioactive compounds are present in great quantity in highly coloured berries [7], with anthocyanins, flavonols, flavanols, phenolic acids and tannins reported as the major phenolic compounds found in foods [8]. Anthocyanins, which are a subclass of flavonoids, are water-soluble pigments responsible for providing red, blue and violet colours present in most plant species [8]. Berries are rich in anthocyanin, which provides pigmentation to fruits and serves as natural antioxidants [9]. The dark red and purple colouration of *C. edulis* is an indication of the presence of anthocyanins in the fruit. Apart from flavonoids, pigmentation in other fruits and vegetables can also be due to the presence of other compounds, such as carotenoids and chlorophylls [10].

*Carissa edulis* berries are richly coloured, as they are red, purple and purplish black when ripe. The fruit is mostly consumed in most rural tribal communities due to its medicinal value and application in different processed commodities, such as beverages, jellies and syrup [11,12]. Several studies conducted on the fruit have reported its antiscorbutic properties [12], therapeutic use in the treatment of anaemia and diabetes [13] as well as its use as an antiplasmodial [14,15], anticonvulsant [16], antiherpetic [17] and antiviral agent [18,19]. *Carissa edulis* fruit can be consumed raw fresh, or it can be processed to other consumed products. However, there exists a scarcity of information in literature on the polyphenolic properties of the fruit, especially at various stages of maturity. Though the fruit is frequently harvested at dark red and purple stages, when its flavour is most desirable as consumers do not usually eat *C. edulis* at other maturation stages, there is thus a need to examine the effect of ripening on the phytochemical, antioxidant and polyphenolic properties of the fruit. This work therefore intends to determine the physicochemical properties and the bioactive compounds present in *C. edulis* fruit at different stage of ripening.

## 2. Results and Discussion

### 2.1. Physicochemical Properties of Carissa edulis Fruit Samples

#### 2.1.1. pH and Total Titratable Acidity (TTA) of *Carissa edulis* Fruit

The analysed data of pH of *C. edulis* are presented in Table 1. An increase in pH value shows that there is a decrease in acidity. The pH of *C. edulis* ranged from 2.85 ± 0.04 (RS1) to 3.10 ± 0.01 (RS3). Significant difference was observed between the pH of samples RS1 and RS3 with the pH increasing as ripening progressed. This observed increase in pH for sample RS3 can be attributed to the degradation of organic acids in the fruit as ripening progressed [20]. The increase in pH during ripening progression is triggered by the decrease in free organic acids as well as the build-up of potassium [20]. Changes in pH can affect enzymatic activity and possibly ionic interactions with calcium, as a change in calcium concentration can affect cell-wall extensibility [21]. The pH of the reference sample was significantly different (*p* < 0.05) from that of RS3 of *C. edulis* fruit. There was, however, no significant difference (*p* < 0.05) between the pH of samples RS1 (2.85 ± 0.04) and RS2 (2.91 ± 0.01) of the *C. edulis* fruit.

The total titratable acid of samples analysed (expressed as citric acid by 0.064 factor (g/100 mL) varied among samples examined. The acidity of *C. edulis* berries ranged from 0.37 ± 0.01 g/100 mL for sample RS1 to 0.27 ± 0.02 g/100 mL for sample RS3 (Table 1). There was no significant difference (*p* < 0.05) between samples RS1 (0.37 ± 0.01 g/100 mL) and RS2 (0.35 ± 0.01 g/100 mL), but both samples differed significantly (*p* < 0.05) from RS3 (0.26 ± 0.02 g/100 mL). The works of Zarei et al. [22] reported a significant decrease (*p* < 0.05) in TTA content at the last stage of ripening in *Punica granatum*. Rubinskiene et al. [23] also reported similar results of reduction in TTA during ripening of black currants fruit. The author reported that the acidity of the black currants fruit at 50% ripeness stage was 5.05%, which reduced to 2.81% at 100% stage of ripeness. Observed decrease in TTA during ripening is attributed to the fact that during ripening, as the concentration of soluble solids build up, the concentration of organic acids decreases due to simple dilution and the utilization of acids in the process of plant respiration [24].

#### 2.1.2. Total Soluble Solids of *Carissa edulis* Fruit

Total soluble solids (^o^Brix) of samples showed significant increase between RS1 and RS3 (Table 1). Significant difference (*p* > 0.05) was recorded among all samples, including the reference sample. The TSS of *C. edulis* fruit samples at RS1 was 9.51 ± 0.21 ^o^Brix, which significantly increased (*p* < 0.05) to 11.12 ± 0.87 ^o^Brix at RS2. The soluble solids again increased significantly (*p* < 0.05) from 11.12 ± 0.09 ^o^Brix in RS2 to 13.51 ± 0.21 ^o^Brix in RS3. The increase of sugars in the berries during ripening is as a result of the storage and breakdown of carbohydrates in the roots and trunk of the vines as well as through the process of photosynthesis. Sucrose produced by photosynthesis is transferred from the leaves to the berries as it is broken down into glucose and fructose molecules upon ripening of the fruit [25].

Rubinskiene et al. [23] reported an increase in soluble solids of black currants ranging from 12.0 ^o^Brix for the 50% ripeness stage to 15.3 ^o^Brix at the 100% ripeness stage. An increase in soluble solids content and decrease in titratable acidity during ripening stages of the fruit was also observed by Rubinskiene et al. [23]. Sample RS3 of *C. edulis* berries recorded the highest concentration of soluble solids (13.51 ± 0.21 ^o^Brix) among the three samples examined. Sample RS3 was observed to also be significantly different (*p* < 0.05) from the soluble solids of SR (13.14 ± 0.07 ^o^Brix).

#### 2.1.3. Sugar/Acid Ratio of *Carissa edulis* Fruit

The concentration of soluble solids/acidity ratio was not static but varied significantly (*p* < 0.05) during fruit development in the samples examined. A decrease in total acidity and an increase in total sugars are important factors in the development of flavour. The sugar/acid ratio has been used as fruit maturity index and has been found to increase with ripening of fruit and decrease as senescence occurs in fruit [26]. The sugar/acid ratio of *C. edulis* ranged from 26.03 to 50.36 (Table 1). Analysed data showed that sample RS3 contained a high TSS and acidity ratio of 50.36 while the TSS/TTA was low for sample RS1 (26.03). Gunduz et al. [27] also reported similar results in cherry fruits. According to the authors, sugar/acid ratio of cherry fruits during different stages of ripening increased as ripening progressed with the reported values for the sugar/acid ratio of cherry fruits occurring at a range of 3.7% to 8.4%.

#### 2.1.4. Colour Properties of *Carissa edulis* Fruit

In the CIELAB system of colour measurement, L* value is a measure of lightness of the samples, and when L* is 0 it means the fruit is black, and when 100 it indicates a diffuse white. The a* value measures redness when positive and greenness when negative, while the b* value measures the blue (negative values) and yellow (positive values) colouration of samples. Apart from its use in the determination of colour characteristics of food samples, the HUNTERLAB colorimeter values have been reported to be used in determining the colour change during ripening or between different stages of ripening of *C. edulis* fruits [8]. Similarly, results for colour properties during fruit maturity have been reported by Celik et al. [28] in cranberry and Özgen et al. [29] in arbutus andrachne fruits.

**Figure 2:**
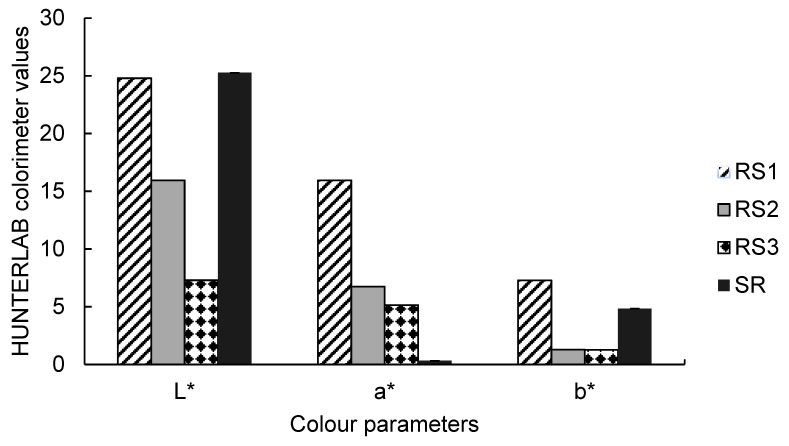
CIELAB colour properties of *Carissa edulis* fruit samples. RS1, first stage of ripening of *C. edulis*; RS2, second stage of ripening of *C. edulis* berries; RS3, third stage of ripening of *C. edulis* berries; SR, standard reference sample (commercial blueberries); L*, lightness of samples; a*, redness when positive and greenness when negative; b*, yellow when positive and blue when negative. Error bars are standard deviation of mean values (*n* = 3).

In this study, the L* value of *C. edulis* ranged from 15.05 ± 0.04 to 24.79 ± 0.78 among the three samples examined (Figure 2). The L* values significantly decreased with ripening progression time, though there was no significant difference between RS2 and RS3. The results showed that sample RS1 was significantly different from all ripening stages among samples analysed. The L* value of the reference sample, 25.26 ± 1.46, was significantly different from sample RS3 of *C. edulis* berries. The a* value of RS3 (5.14 ± 0.19) was significantly different (*p* < 0.05) from RS2 (6.74 ± 0.13). The b* value of *C. edulis* ranged from 7.29 ± 0.49 to 1.27 ± 0.09 among the three samples. The b* value of RS1 (7.29 ± 0.48) was significantly different (*p* < 0.05) from RS2 (1.24 ± 0.21) and RS3 (1.27 ± 0.09). The significantly low b* values were expected as the berries were more red than yellow.

These observations show that the ripening progression had a high impact on colour change of *C. edulis* fruits. Eichholz et al. [30] reported that colour changes during fruit ripening implies both synthesis and degradation of the plant pigments, including chlorophyll, carotenoids as well as flavonoids. As the berries ripen, the concentration of phenolic compounds, such as anthocyanins, replaces the green colour of chlorophyll in the berries, which makes them purple instead. Colour change in fruit samples is as a result of pigments which were always present in the fruit and becomes visible when chlorophyll is degraded [31].

### 2.2. Phytochemical Properties of Carissa edulis Fruit Samples

#### 2.2.1. Total Phenolic Content (TPC) of *Carissa edulis* Fruit

The TPC of samples was determined using the Folin–Ciocalteu reagent on aqueous extracts of *C. edulis* berries. Results of TPC ranged from 5.90 ± 0.41 mg GAE/g to 6.81 ± 0.02 mg GAE/g among the three *C. edulis* samples examined (Table 2). The extracts of all four samples had higher concentrations of TPC at the maturity stage RS1. Subsequently, there was an observed decrease in TPC during colour break and ripening, particularly at the first stage in unripe berries. The TPC of *C. edulis* samples was significantly different (*p* < 0.05) during the different stages of ripening, with the TPC of *C. edulis* fruit samples decreasing with ripening stage progression. At RS1, TPC was 6.81 ± 0.02 mg GAE/g, which decreased to 5.90 ± 0.41 mg GAE/g at RS2 and then increased to 6.71 ± 0.13 mg GAE/g at the final stage of ripening (RS3). These results can be compared to those of Castrejón et al. [32] who studied the phenolic profile and antioxidant activity of highbush blueberries during fruit maturation and ripening. The authors reported that TPC at the first stage of ripening had higher concentration of 42 mg GAE/g, which reduced to 30 mg GAE/g at the second stage of ripening and was further reduced to 19 mg GAE/g at the third stage of ripening.

Kalt et al. [33] reported a decrease in TPC at a ripe stage of fruit maturity for bush blueberries. The authors reported a TPC of 12.5 ± 2.10 mg GAE/g DW for high bush blueberries at 50% ripeness stage, with the TPC increasing to 18.5 ± 2.34 mg GAE/g DW at 100% ripeness stage. As observed by Mäkilä [34], reported decrease in TPC in fruits may be due to environmental factors, such as sunlight and room temperature, during sample analysis as total phenolics determination is very sensitive to sunlight and high temperature. Phenolic compounds are generally sensitive to both temperature and light, with Mäkilä [34] reporting a light induced conversion of (*E*)-p-coumaric acid derivatives into the corresponding (*Z*)-isomers. Similarly, Zheng and Wang [35] reported an increase in phenolic content at high temperatures of growth and a decrease during lower temperatures of growth.

Wang and Lin [36] observed that the content of total phenolics increased in black and red raspberry from pink to the ripe stage, whereas for other berry species, such as strawberry and blackberry, the less ripe berries had higher contents of total phenolics than the fully ripe berries. Similarly, Shin et al. [37] reported decreasing TPC in strawberries with enhanced ripening. Such variations in the TPC are mainly due to differences in genotypes of the cultivar, although several factors such as temperature, light, and analytical conditions are known to be responsible for these dissimilarities [38,39]. The works of Eichholz et al. [30] suggested that during ripening of highbush blueberry, there was a shift in the pool of total polyphenols towards anthocyanin synthesis and an overall decline in the content of further phenolic compounds. However, additional quantitative studies of individual phenolic compounds appear to be needed for further clarification of the mechanisms responsible for the observed variations [40].

In the work done by Kutz [41], phenolic antioxidants were reported to exhibit a weak absorption tail extending well above 300 nm, which makes them easily transformed when they are irradiated with terrestrial sunlight’s wavelength. As a result, phenolic antioxidants are not very effective UV stabilisers. Light, UV-radiation, fungal infection, interaction with microorganisms or wounding affects the phenolic profiles of plant tissues including the flavonoid biosynthesis [42,43]. These factors imply stress in plants and thus results in an accumulation of phenolic compounds as a plant defence response [43]. Hence, during the analysis of *C. edulis* fruit samples, fruit extract from samples were kept in a brown box for 30 min to avoid degradation of bioactive compounds.

#### 2.2.2. Total Flavonoids of *Carissa edulis* Fruit

As reported by Harborne and Williams [44], flavonoids account for 60% of total dietary phenolic content. Flavonoids are phenolic compounds, which are very effective antioxidants [45]. The predominant flavonoids found in berries and red grapes are anthocyanins and flavonols, which are almost exclusively present in their glycosylated forms [30]. The total flavonoids content (TFC) of samples obtained in this study ranged from 5.09 ± 0.04 mg CE/g to 5.95 ± 0.76 mg CE/g (Table 2) with the TFC of *C. edulis* samples increasing with ripening stage progression. Total flavonoids were found to be significantly higher (*p* < 0.05) at RS3 (5.92 ± 0.03 mg CE/g) among samples examined. Results of the study also showed that there was no significant difference (*p* > 0.05) between RS1 (5.09 ± 0.04 mg CE/g) and RS2 (5.92 ± 0.03 mg CE/g), yet RS3 (5.95 ± 0.76 mg CE/g) was significantly different (*p* < 0.05) from RS1 and RS3. Sample SR with a flavonoid concentration of 6.31 ± 0.27 mg CE/g and used as standard in this study was significantly different from sample RS3 at the ripe stage of maturity.

Flavonoids concentration of *C. edulis* berries used in this study increased with ripening stage progression. There is evidence that flavonoid biosynthesis is associated with the development stages of the fruit [32]. The enzyme activities are controlled in response to different developmental and environmental conditions.

#### 2.2.3. 2,2 diphenyl-1-picryl-hydrazyl (DPPH) Radical Scavenging Activity of *Carissa edulis* Fruit

The DPPH radical scavenging assay was employed in evaluating the antioxidant activity of plant extracts due to its effectiveness [46]. Antioxidants are compounds that are capable of donating electrons of hydrogen atom to inhibit a free radical reaction [8]. An antioxidant effect is observed by scavenging free radicals that are involved in slowing or inhibiting oxidative stress.

The DPPH scavenging activity of sample RS1 (20.24 ± 0.27 mmol TE/g) was significantly higher (*p* < 0.05) than scavenging activities of RS2 (19.09 ± 0.02 mmol TE/g) and RS3 (18.36 ± 0.12 mmol TE/g). There was a significant difference (*p* < 0.05) between the three different stages of ripening of *C. edulis* berries. Generally, the DPPH scavenging activities of all *C. edulis* samples decreased with the ripening stage progression, with sample RS3 recording a significantly least antioxidant activity of 18.36 ± 0.12 mmol TE/g among all samples examined. The reference sample SR exhibited an antioxidant activity of 20.26 ± 0.56 mmol TE/g, which was not significantly different from sample RS1. Gunduz et al. [27], who conducted a study on the antioxidant, physical and chemical characteristics of cornelian cherry fruits (*Cornus mas*) at different stages of ripeness, reported that the antioxidant activity of the cornelian cherry fruits ranged from 55.0–7.8 μmol TE g^−1^ FW and this decreased with the ripening progression. At the first stage of ripening of the cornelian fruits (with light yellow colouration), the fruits contained significantly higher antioxidant activity (55.0 μmol TE g^−1^ FW) than the last stage of ripening (with dark red colouration), which contained significantly lower antioxidants activity (7.8 μmol TE g^−1^ FW).

#### 2.2.4. Polyphenolic Profile of *Carissa edulis* Fruit

**Figure 3:**
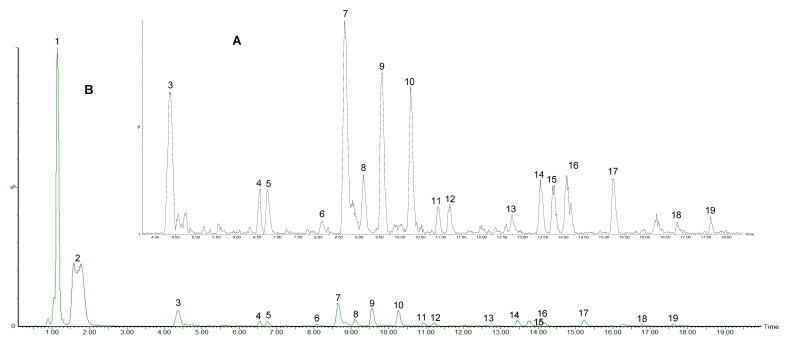
TIC chromatograms of *Carissa edulis* fruit samples at ripening stage 1. (**A**) Zoomed in chromatogram > 4 min; (**B**) full chromatogram.

**Figure 4:**
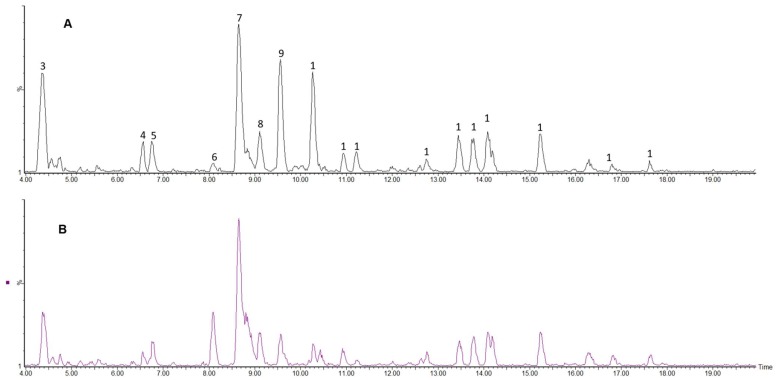
Comparison of TIC chromatograms at different ripening stage: (**A**) Ripening stage 1 (RS1) and (**B**) ripening stage 2 (RS2) (MS^E^ F1: low-energy CID).

Phenolic compounds in *C. edulis* fruit were analysed by liquid chromatography-mass spectrometry (LC-MS) using a Waters Acquity ultra-performance liquid chromatography (UPLC) with photo diode array (PDA) detector connected to a Waters Synapt G2 quadrupole time-of-flight (QTOF) mass spectrometer (Waters, Milford, MA, USA). Detected compounds were tentatively characterised by means of high-resolution MS data together with the interpretation of the observed MS^E^ and UV spectra in comparison with those in literature and online databases. Several compounds were identified from *C. edulis* fruit samples, as shown in Figure 3 and Figure 4. These compounds together with their retention times, mass errors, molecular ions and their tentative names are presented in Table 3. Polyphenolic compounds obtained from samples of *C. edulis* fruits used in this study could be classified primarily as phenolic acids and flavonoids. Other compounds not classified as polyphenolic compounds were also identified in the fruit samples.

It is well known that flavonoid biosynthesis is closely related to the developmental stages of the fruit [32,33,47,48]. As reported by Halbwirth et al. [48], two distinct flavonoid enzyme activity peaks exist during ripening: The first corresponding to the production of flavonols and phenolic acids at the early stage of ripening accompanied by a second biosynthesis peak involving a shift in enzyme pathways towards the production of anthocyanins during berry ripening. A total of 19 compounds were found to be present in the first stage of ripening (peaks **1**–**19**). Peaks **1**, **4**, **5**, **7**, **8** and **18** were identified as quinic acid (*m*/*z* 191.0181, λ: 280), protocatechuoyl-hexose (*m*/*z* 315.0715, λ: 306), neochlorogenic acid (*m*/*z* 353.0854, λ: 284), chlorogenic acid (*m*/*z* 353.0876, λ: 191), cryptochlorogenic acid (*m*/*z* 353.0886, λ: 284) and dicaffeoylquinic acid (*m*/*z* 515.1204, λ: 329), as seen in Table 3. Peaks **13** (a quercetin diglycoside) and **16**, a quercetin monoglycoside, showed molecular ion at *m/z* 595.132 and 463.0867 with formulas C_26_H_27_O_16_ (3.5) and C_21_H_19_O_12_ (0.4). Both peaks **13** and **16** were identified as quercetin-3-*O*-glucosyl-xyloside and quercetin-3-*O*-glucoside. Furthermore, peaks **14** and **15**, which are quercetin diglycosides and showed molecular ion at *m*/*z* 609.1467 and 609.147 with formulas C_27_H_29_O_16_ (3.8) and C_27_H_29_O_16_ (2.6), were identified as quercetin-3-*O*-robinobioside and quercetin-3-*O*-rutinoside. Peaks **9**, **10**, **12** and **17** with molecular ion at *m/z* 577.1357 (C_30_H_25_O_12_, 2.6), 289.0695 (C_15_H_13_O_6_, 1.7), 865.1946 (C_45_H_37_O_18_, −3.9) and 607.1327 (C_27_H_27_O_16_, −0.5) were identified as procyanidin dimer, catechin, procyanidin trimer and quercetin-3-OH-3-methylglutaryl-glucoside, respectively.

Phenolic acids present in *C edulis* fruit samples include: Quinic acid, protocatechuoyl-hexose, neochlorogenic acid, chlorogenic acid, cryptochlorogenic acid and dicaffeoylquinic acid. Authors Lachowicz et al. [10] reported the presence of neochlorogenic, chlorogenic, cryptochlorogenic and protocatechuic acid among other phenolic acids in Saskatoon berry genotypes grown in central Poland. Similarly, Brito et al. [49] reported the presence of chlorogenic and neochlorogenic acid in their work on anthocyanin characterization, total phenolic quantification and antioxidant features of some Chilean edible berry extracts. In *C. edulis* fruit samples examined in this study, concentration of chlorogenic acid was seen to be high at both stages of ripening with the concentration of chlorogenic acid ranging from 215.87 ± 7.10 (RS1) to 215.15 ± 16.03 μg/g (RS2). It could be inferred that *C. edulis* at different stages of ripening is a good source of chlorogenic acid. Gibson et al. [50] reported a high concentration of chlorogenic acid in lowbush blueberries during the stages of maturity. The authors further stated that the first stage of maturity on lowbush blueberries contained 307 ± 32 mg/100 g DW of chlorogenic acid while the second stage of maturation contained 200 ± 25 mg/100 g DW of chlorogenic acid concentration. As observed, concentration of phenolic acids varied at different stages of ripening with its occurrence differing as ripening progressed.

**Figure 5:**
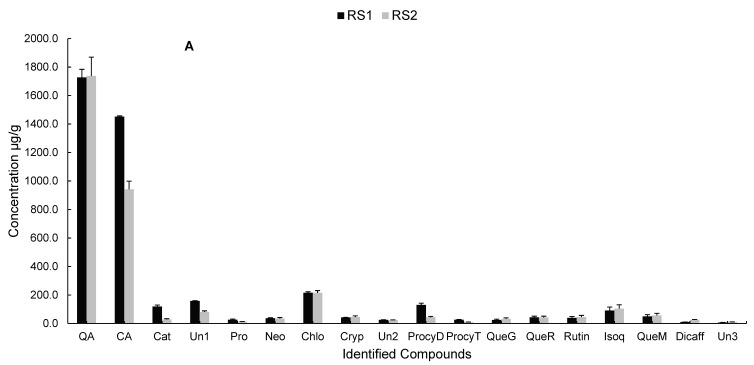

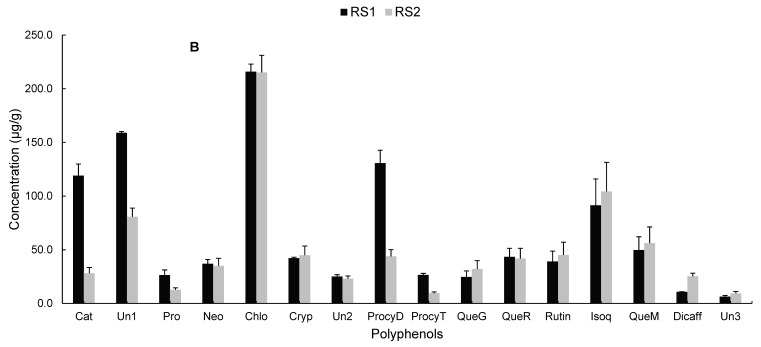
Compounds present in *Carissa edulis* fruit. (**A**) Identified compounds in *C. edulis* fruit; (**B**) polyphenolic content of *Carissa edulis* fruit. RS1, first stage of ripening of *C. edulis*; RS2, second stage of ripening of *C. edulis* berries; QA, quinic acid; CA, citric acid; Cat, catechin; Un1, unknown compound 1; Pro, protocatechuoyl-hexose; Neo, neochlorogenic acid; Chlo, chlorogenic acid; Cryp, cryptochlorogenic acid; Un2, unknown compound 2; ProcyD, procyanidin dimer; ProcyT, procyanidin trimer; QueG, quercetin-3-*O*-glucosyl xyloside; QueR, quercetin-3-*O*-robinobioside; Isoq, isoquercitrin (quercetin-3-*O*-glucoside); QueM, quercetin-3-OH-3-methylglutaryl-glucoside; Dicaff, dicaffeoylquinic acid; and Un3, unknown compound 3. Error bars are standard deviation of mean values (*n* = 3).

Flavonoids identified in *C. edulis* samples examined include: procyanidin dimer, procyanidin trimer, catechin, quercetin-3-*O*-glucosyl-xyloside, quercetin-3-*O*-robinobioside, quercetin-3-*O*-rutinoside (rutin), quercetin-3-*O*-glucoside (isoquercitrin) and quercetin-3-OH-3-methylglutaryl-glucoside. Jurikova et al. [51] reported the presence of rutin, quercetin monoglycosides (quercetin-3-*O*-glucoside and quercetin-3-glucosyl-xyloside) and quercetin diglycosides (quercetin-3-*O*-robinobioside and quercetin-3-rutinoside) in a review on flavonoid profile of Saskatoon berries and their health promoting effects while Brito et al. [49] further confirmed the presence of isoquercitrin, quercetin and rutin in their work done on some Chilean edible berry extracts. Other flavonoids, such as procyanidin dimer, catechin, caffeoylquinic acid derivatives and quercetin-3-*O*-robinobioside, were identified in chokeberry juice and dried fruits by Oszmianski and Lachowicz [52]. Catechin was identified in *C. edulis* berries at different stages of ripening and at varying concentrations. At RS1, *C. edulis* contained higher concentration of catechin (118.97 ± 10.78 μg/g), while *C. edulis* at RS2 contained catechin concentration of 28.24 ± 5.31 μg/g (Figure 5a,b). In the works of Sun et al. [53], catechin at a concentration of 48.9 mg/kg was identified in grapes at maturation stage of development. The author equally reported identification of catechin at veraison stage with a concentration of 28.7 mg/kg. Similarly, Stanila et al. [54] also reported catechin as the most abundant polyphenolic compound in *Rosa canina* wild berries.

Procyanidin dimer was also identified in large concentrations of 130.66 ± 12.05 μg/g in RS1 and 43.91 ± 6.23 μg/g in sample RS2. Procyanidin is a phenolic compound with four to eight bonds that possesses the ability to act as free radical scavenger. Sample RS1 recorded a higher procyanidin dimer concentration when compared to sample RS2. This shows that ripening stage progression affected the procyanidin dimer concentration of *C. edulis* samples. Rutin, another flavonoid identified in samples RS1 and RS2 of *C. edulis* samples, examined recorded a concentration of 39.15 ± 9.67 to 45.24 ± 11.85 μg/g for samples RS1 and RS2, respectively. A similar result was observed by Gibson et al. [50], who reported a decrease in rutin concentration during different maturation stages in lowbush blueberries. It must be noted, however, that there was an observed increase in rutin concentration between samples RS1 (39.15 ± 9.67 μg/g) and RS2 (45.24 ± 11.85 μg/g) as ripening progressed.

Apart from polyphenols identified in *C. edulis* fruit samples, other plant nutrients, such as citric acid and some unknown compounds not grouped as polyphenols, were observed in the fruit samples examined.

## 3. Materials and Method

### 3.1. Fruit Samples

*Carissa edulis* fruits used for this study were harvested at various stage of ripeness at Ha-Mashau Limpopo Province, South Africa. Upon harvest, fruits were grouped according to their state of maturity and ripeness: First stage of ripening (red ripe), RS1; second stage of ripening (purple ripe), RS2; and last stage of ripening (purplish black ripe), RS3. Commercial blueberries purchased at Shoprite Thohoyandou, South Africa, were used as standard reference sample (SR). *Carissa edulis* fruit samples at various stage of senescence and in their unprocessed fresh state were used for determination of the various physicochemical properties. Samples were then stored at freezing temperature of −20 °C for further use.

### 3.2. Preparation of Plant Extracts

Plant extracts were prepared by refluxing 2 g of crushed *C. edulis* samples with 20 mL of methanol containing 1% HCl for 2 h at 60 ± 5 °C [55]. The mixtures were centrifuged (5000 rpm, 20 min) and the supernatants separated and used for analysis of total phenolic content, total flavonoid content and antioxidant activity.

### 3.3. 2,2-diphenyl-1-picrylhydrazyl (DPPH) Radical Scavenging Activity

The DPPH radical scavenging activity was determined according to the method of De Ancos et al. [56] with some modifications. An aliquot (10 µL) of the acidified methanolic extract was mixed with distilled water (90 µL) and 3.9 mL of methanolic 0.1 mM DPPH solution. The mixture was thoroughly vortexed and kept in the dark for 30 min, and the absorbance was read at 515 nm. A calibration curve was prepared using a standard solution of Trolox (R^2^ = 0.9633) and results were expressed as mmol of Trolox equivalent (TE) per g of the sample. All samples were analysed in triplicate.

### 3.4. Total Phenolic Content Determination 

Total phenolic content was determined by the Folin–Ciocalteu method, a colorimetric assay based on procedures described by Singleton et al. [57]. Briefly, 0.1 mL of the acidified methanolic extract was mixed with 5 mL distilled water in a 50 mL volumetric flask. Folin–Ciocalteu’s reagent (1:2 dilution with water, 2.5 mL) and 7.5 mL 15% sodium carbonate solution were added, mixed thoroughly, made up to 50 mL and allowed to react for 30 min. The absorbance of the reaction mixture was read at 760 nm with a spectrophotometer. A calibration curve was prepared using a standard solution of gallic acid (R^2^ = 0.995) and result was expressed as mg of gallic acid equivalent (GAE) per g of the sample. All samples were analysed in triplicate.

### 3.5. Total Flavonoids Determination

Total flavonoid content was determined spectrophotometrically according to Zhishen et al. [58]. A 0.1 mL extract was mixed with 4.9 mL distilled water and 0.3 mL (5% *w*/*v*) NaNO_2_ was added. After 5 min, 0.3 mL (10% *w*/*v*) AlCl_3_ and at 6 min, 2 mL 1 M NaOH were added, with the total volume immediately made up to 10 mL using distilled water. The mixture was vortexed and the absorbance read at 510 nm. A calibration curve was prepared using catechin hydrate standard (R^2^ = 0.9317) and the result expressed as mg catechin equivalents per g of the sample. All samples were analysed in triplicate.

### 3.6. Determination of pH and Titratable Acidity

The pH of samples was determined in triplicate at room temperature using a Fisher Accumet, Model 15, pH Meter (Fisher Scientific, Edmonton, AB, Canada). Three-point calibration was accomplished employing pH 7.0, 4.0 and 2.0 buffers [4,8].

The method of AOAC 942.15 [59] was used in the determination of titratable acidity. Approximately 10 g of crushed *C. edulis* berries were diluted to 250 mL with distilled water and titrated with 0.1N NaOH using 0.3 ± 0.1 mL of phenolphthalein for each 100 mL of solution to pink end persisting for 30 s. Acidity was reported as mL 0.1 N NaOH per 100 mL. Total titratable acidity was expressed as citric acid by 0.064 factor (g/100 mL). All analyses were carried out in triplicate.

### 3.7. Determination of Soluble Solids (^o^Brix)

Total soluble solids of samples were determined spectrophotometrically according to Green [8] with some modifications [4]. Soluble solids were expressed as ^o^Brix for obtained data from samples examined. The fruits were crushed using pestle and mortar, and soluble solids were determined on the crushed berries using a Leica Auto Abbe refractometer, Model 10,504 (Leica Inc., Buffalo, NY, USA) with temperature compensation. Fruit samples were analysed in triplicates.

### 3.8. Determination of Colour Parameters 

The colour of *C. edulis* was measured using a colorimeter (ColourFlex, HunterLab, Hunter Associates Laboratory, Inc., Reston, VA, USA) according to the method of AOAC 955.23 [57]. The colorimeter was calibrated with a standard white (L* = 93.71, a* = –0.84 and b* = 1.83). The berries were transferred to a glass sample cup (Hunter Associates) and placed over the analysis port on the colour meter. A black cover cup was placed over the sample, and the CIELAB L*, a* and b* values were measured. Colour was expressed as *L*-value (lightness/darkness), *a*-value (redness/greenness) and *b*-value (yellowness/blueness). Samples were analysed in triplicate.

### 3.9. Liquid Chromatography Coupled to Diode Array Detection and Electrospray Ionisation Mass Spectrometry (LC-DAD-ESI-MS) Analysis of Phenolic Compounds in Carissa edulis Fruit

A total of 10–15 fruit samples were homogenised and macerated using a mortar and pestle. To the crushed *C. edulis* samples, 5 g was removed and placed in sample bottles. A total of 20 mL of 50% methanol and 1% formic acid was then added to the 5 g crushed sample. The mixture was vortexed and allowed to stand overnight for extraction. The solution was then sonicated for 30 min and 2 mL of the aliquot centrifuged at 12,000 rpms for 5 min. From the centrifuged solution, 1 mL of the fruit sample was placed in vials, sealed in preparation for liquid chromatographic analysis. Liquid chromatography mass spectrometry (LC-MS) analysis was conducted on a Waters Synapt G2 quadrupole time-of-flight mass spectrometer (Waters, Milford, MA, USA). The instrument was connected to a Waters Acquity ultra-performance liquid chromatography (UPLC) and Acquity photo diode array (PDA) detector. Ionisation was achieved with an electrospray source using a cone voltage of 15 V and capillary voltage of 3 kV using negative mode for determination of phenolic compounds. Data were acquired in MS^E^ mode, which involves a low collision energy scan followed by a high collision energy scan to acquire both molecular ion and fragmentation data in one run.

Desolvation gas used was nitrogen at a flow rate of 650 L/h and desolvation temperature of 275 °C. Aqueous formic acid 0.1% (*v*/*v*) and acetonitrile were used as mobile phases A and B, respectively. Separation was carried out in 29 min under the following conditions: 0 min, 0% B; 22 min, 28% B; 22.5 min, 40% B; 23 min, 100% B; 25 min, 0% B, with the initial conditions held for 4 min as a re-equilibration step. The separations were conducted on a Waters Acquity BEH C18 column (2.1 × 100 mm, 1.7 μm particle size), with injection volume of 3 μL at flow rate of 0.4 mL/min. Epicatechin standard obtained from Sigma-Aldrich, South Africa, was used for relative quantification of phenolic compounds in *C. edulis* fruit with samples extracted in triplicate and injected three times. Detected compounds were tentatively characterised by means of MS data together with the interpretation of the observed MS^E^ spectra in comparison with those in literature and online databases, such as Metlin, ChemSpider and MassBank. Obtained data were expressed as microgram epicatechin per gram of sample.

### 3.10. Statistical Analysis

The fruit samples were analysed in triplicates for each determined fruit assay. Statistical analysis was performed using one-way analysis of variance (ANOVA) and means of triplicate determinations separated using Duncan multiple range test (*p* < 0.05). The results of analysis were expressed as mean values ± standard deviation (SD) and all statistical analysis performed using IBM SPSS Statistics for windows version 24 (IBM Corp., Armonk, NY, USA).

## 4. Conclusions

Obtained results clearly demonstrate that the phenolic content, antioxidant and physicochemical properties of *C. edulis* fruits are affected by ripening stages. Significant variability was observed for physicochemical (colour, total solid solubility and pH) antioxidant and phenolic properties among all three stages of ripening of *C. edulis* fruit samples. Recorded antioxidant activities, total phenolics and flavonoid content as well as individual polyphenols with varying concentrations were equally reported among ripening stages in fruit samples examined. Although ripe *C. edulis* berries are generally more edible at the final stage of ripening, greater antioxidant activity was observed at the first stage of ripening while total flavonoid content increased with progression in ripening. A total of 19 compounds—14 phenolics, 1 organic and 4 unknown compounds—were identified in *C. edulis* berries used in this study with the identified polyphenolic compounds mainly of the phenolic acids and flavonoid groups. Citric acid was the organic acid identified in fruit samples with the other unknown compounds. This study thus provides, for the first time, information regarding polyphenolic compounds present in *C. edulis* fruit and at different stages of ripening of the berries. More studies on the phenolic profiles during ripening of different genotypes in relation to colour, flavour, acidity and other attributes need be investigated.

## Figures and Tables

**Table 1:** pH, total acidity and total soluble solids of *Carissa edulis* fruit samples.

Samples	pH	TTA (g/100 mL)	TSS (^o^Brix)	TSS/TTA
RS1	2.85 ± 0.04 ^a^	0.37 ± 0.01 ^c^	9.51 ± 0.21 ^a^	26.03
RS2	2.91 ± 0.01 ^a^	0.35 ± 0.01 ^c^	11.12 ± 0.08 ^b^	31.95
RS3	3.10 ± 0.01 ^b^	0.27 ± 0.02 ^b^	13.51 ± 0.21 ^d^	50.36
SR	3.32 ± 0.07 ^c^	0.21 ± 0.02 ^a^	13.14 ± 0.07 ^c^	61.55

Means in the same column with the same superscript letter ^(a, b, c, d)^ are not significantly different (*p* > 0.05). RS1 = first stage of ripening of *C. edulis* fruit; RS2 = second stage of ripening of *C. edulis* fruit; RS3 = third stage of ripening of *C. edulis* fruit; SR = standard reference sample (commercial blueberries); pH = potentia hydrogenii; TTA = total titratable acid; ^o^Brix = soluble solids; TSS/TTA = sugar/acid ratio. Values are means ± standard deviation (*n* = 3).

**Table 2:** Total phenolic and flavonoid content of *Carissa edulis* fruit samples.

Samples	TPC (mg GAE/g)	TFC (mg CE/g)	DPPH (mmol TE/g)
RS1	6.81 ± 0.02 ^b^	5.09 ± 0.04 ^b^	20.24 ± 0.27 ^c^
RS2	5.90 ± 0.41 ^a^	5.92 ± 0.03 ^b^	19.09 ± 0.02 ^b^
RS3	6.71 ± 0.13 ^c^	5.95 ± 0.76 ^a^	18.36 ± 0.12 ^a^
SR	7.21 ± 0.23 ^c^	6.31 ± 0.27 ^b^	20.26 ± 0.56 ^c^

Means in the same column with the same superscript letter ^(a, b, c)^ are not significantly different (*p* > 0.05) for *C. edulis*. RS1 = first stage of ripening of *C. edulis*; RS2 = second stage of ripening of *C. edulis*; RS3 = third stage of ripening of *C. edulis*; SR = standard reference sample (commercial blueberries); TFC = total flavonoids content; TPC = total phenolic content; DPPH = 2,2 diphenyl-1-picryl-hydrazyl; GAE = gallic acid equivalent; CE = catechin equivalent. Values are means ± standard deviation (*n* = 3).

**Table 3:** Compounds identification in *Carissa edulis* fruit.

Peak No.	Rt (min)	[M − H]^−^ (*m*/*z)*	[M − H]^−^ Formula	Error (ppm)	MS^E^ Fragments (*m*/*z)*	UV (nm)	Tentative Identification	Classification
**1**	1.16	191.0539	C_7_H_11_O_6_	2.6	85	264	Quinic acid	Phenolic acids (Cyclic polyol)
**2**	2.17	191.0181	C_6_H_7_O_7_	0.6	155, 127, 111	280	Citric acid	Organic acid
**3**	4.35	309.1188	C_12_H_22_O_9_	−1.0	309, 129	Weak	Unknown	
**4**	6.56	315.0715	C_13_H_15_O_9_	−0.3	153, 109	306	Protocatechuoyl-hexose	Phenolic acids
**5**	6.75	353.0854	C_16_H_17_O_9_	1.7	191, 179, 135	325	Neochlorogenic acid (3CQA)	Phenolic acids
**6**	8.09	175.0605	C_7_H_11_O_5_	−2.9	115	Weak	Unknown	
**7**	8.65	353.0876	C_16_H_17_O_9_	−2.5	191	325	Chlorogenic acid (5CQA)	Phenolic acids
**8**	9.10	353.0886	C_16_H_17_O_9_	0.8	191, 179, 173, 135	325	Cryptochlorogenic acid (4CQA)	Phenolic acids
**9**	9.56	577.1357	C_30_H_25_O_12_	2.6	407, 289	279	Procyanidin dimer	Flavonoid (Proanthocyanidin/condensed tannin)
**10**	10.26	289.0695	C_15_H_13_O_6_	1.7	245, 203, 179, 137, 125	278	Catechin	Flavonoid (Flavan-3-ol)
**11**	10.93	439.1859	C_18_H_31_O_12_	−1.8	408, 289, 161, 125	278	Unknown	
**12**	11.21	865.1946	C_45_H_37_O_18_	−3.9	695, 575, 407, 289, 161	278	Procyanidin trimer	Flavonoid (Proanthocyanidin/condensed tannin)
**13**	12.75	595.132	C_26_H_27_O_16_	3.5	300, 271, 255	349	Quercetin-3-*O*-glucosyl-xyloside	Flavonoid (Flavonol-glycoside)
**14**	13.44	609.1467	C_27_H_29_O_16_	3.8	301, 300, 271, 255	351	Quercetin-3-*O*-robinobioside	Flavonoid (Flavonol-glycoside)
**15**	13.75	609.147	C_27_H_29_O_16_	2.6	301, 300, 271, 255	351	Quercetin-3-*O*-rutinoside (rutin)	Flavonoid (Flavonol-glycoside)
**16**	14.06	463.0867	C_21_H_19_O_12_	0.4	300, 271, 255	353	Quercetin-3-*O*-glucoside (isoquercitrin)	Flavonoid (Flavonol-glycoside)
**17**	15.24	607.1327	C_27_H_27_O_16_	−0.5	505, 463, 300, 271	351	Quercetin-3-OH-3-methylglutaryl-glucoside	Flavonoid (Flavonol-glycoside)
**18**	16.82	515.1204	C_25_H_23_O_12_	2.7	191, 179, 173, 135	329	Dicaffeoylquinic acid	Phenolic acids
**19**	17.61	1381.4824	C_60_H_85_O_36_	−0.3	869, 827, 511, 409	277	Unknown	

Rt, retention time.
